# Arteriolar vs. valvular thrombosis: Pick your evil!

**DOI:** 10.1186/s12959-018-0175-3

**Published:** 2018-08-29

**Authors:** Abeer Berry, George Degheim, Souheil Saba

**Affiliations:** St John Providence-Providence Park Hospitals, 19001 W. 9 Mile Rd, Southfield, MI 48075 USA

## Abstract

**Background:**

Anticoagulation therapy for mechanical prosthetic valves is limited to vitamin K antagonists, unfractionated heparin and low-molecular-weight-heparin. Other forms of anticoagulation are either contraindicated or have not been well studied. Hence, anticoagulation for preexisting mechanical valves is controversial if vitamin K antagonists are contraindicated. We present a case involving an end-stage-renal disease patient with both mitral and aortic mechanical valves who developed warfarin-induced calciphylaxis.

**Case presentation:**

A 72-year-old male with history of end-stage renal disease, chronic atrial fibrillation and rheumatic heart disease status post mitral and aortic valve replacements presented with complaints of left thigh erythema with skin induration. Despite multiple antibiotic regimens for presumed cellulitis, the skin lesions progressed to necrotic ulcers. A biopsy revealed evidence of calciphylaxis; a lethal condition typically associated with renal disease. The patient was on warfarin for anticoagulation of his mechanical heart valves as well as prophylactically for atrial fibrillation. Warfarin contributes to the development of calciphylaxis and needed to be exchanged to avoid progression of the ulceration. The only other acceptable option for long-term anticoagulation was subcutaneous unfractionated heparin but this approach was not taken. The patient suffered from further sequelae of calciphylaxis and eventually expired.

**Conclusion:**

Calciphylaxis is a rare, serious disorder that presents with skin ischemia and necrosis mainly in end-stage renal disease patients. The pathogenesis and treatment are poorly understood and the prognosis remains grave. It is proposed that certain medications, including warfarin, contribute to its evolution. The optimal anticoagulation therapy in those with concomitant warfarin-induced calciphylaxis and mechanical valves is undetermined. Further studies are essential to establish new anticoagulation regimens in these devastating circumstances.

## Background

Valvular heart disease is a prominent condition that affects millions of patients. When symptoms or structural heart disease develop due to a malfunctioning valve, it may be replaced with a prosthetic valve. The prosthesis improves quality of life by reducing symptoms and increasing lifespan. However, this does not come without risk. There are serious complications involved with the prosthesis depending on what type of prosthetic valve is implanted. Prosthetic valves may lead to embolic events, infective endocarditis, bleeding and valve obstruction. The most common complications of mechanical heart valves involve anticoagulation-related problems and thromboembolism.

Antithrombotic therapy for prosthetic heart valves is dependent on the type of valve. Mechanical prosthetic valves require lifetime anticoagulation with or without antiplatelet therapy. The current options for anticoagulation with mechanical heart valves are limited. Vitamin K antagonists (VKAs), low molecular weight heparin (LMWH) and unfractionated heparin (UFH) are the only approved agents at this time [1]. Hence, anticoagulation for preexisting mechanical valves becomes a dilemma if VKAs and LMWH are not recommended in certain patient populations. We present a case involving an end-stage-renal disease (ESRD) patient with both mitral and aortic mechanical valves who developed VKA-induced calciphylaxis.

## Case report

A 72-year-old male with a past medical history of ESRD, chronic atrial fibrillation (AF) and rheumatic heart disease (RHD) status post mechanical mitral and aortic valve replacements presented to his primary care doctor complaining of left thigh erythema with skin induration. The patient had been on warfarin therapy for anticoagulation of his mechanical heart valves as well as prophylactically for underlying AF for greater than 20 years. He was initially diagnosed with cellulitis and treated accordingly with antibiotics. Despite multiple antibiotic regimens, the skin lesions did not improve and instead progressed into painful, necrotic ulcers. The lesions were evaluated by his nephrologist 2 months later who deemed cellulitis to be a misdiagnosis and recommended a biopsy of the skin lesions. The biopsy revealed pathology consistent with calciphylaxis, a lethal disease typically associated with ESRD.

The disease is known to be exacerbated by certain medications including warfarin, vitamin D analogs, calcium-based binders and glucocorticoids [2]. Other risk factors in ESRD patients include diabetes, hyperphosphatemia, obesity, hyperparathyroidism and hypercalcemia. The patient was on warfarin therapy and vitamin D analogs. He was also taking sevelamer, a non-calcium-containing phosphate binder, to prevent hyperphosphatemia.The vitamin D supplementation was discontinued but the cessation of warfarin was controversial.

Since warfarin contributes to the development of calciphylaxis, it should have been exchanged for another form of anticoagulation to avoid progression of the non-healing, necrotic ulcerations. It was not accomplishable in this circumstance since LMWH is not Food and Drug Administration (FDA) approved in ESRD and is associated with serious bleeding and the need for frequent dose adjustments and monitoring [3]. The only other option for long-term anticoagulation was UFH administered subcutaneously but this approach was not taken. It is difficult to maintain therapeutic levels with UFH as it requires massive doses to do so. Because it was believed that the risk of two mechanical valve thromboses outweighed the risk of the discontinuation of anticoagulation, the warfarin was continued. Unfortunately, the lesions progressed over 1 year and the patient suffered from the sequelae of calciphylaxis despite the addition of sodium thiosulfate infusions to each hemodialysis session. With the advancement of the ischemic ulcerations, the patient developed superinfections and debridement was not sufficient to control the sepsis. He eventually expired due to septic shock.

## Discussion

Prosthetic heart valves provide symptomatic relief and reduce morbidity and mortality in patients with valvular heart disease. They are associated with their own complications including thromboembolism and bleeding especially in the setting of mechanical prosthetic valves. Mechanical valves require lifetime anticoagulation and the agents approved for this indication are restricted. Warfarin, LMWH and UFH are the only three anticoagulants that can be utilized with mechanical prostheses [1]. Dabigatran is the sole direct oral anticoagulant (DOAC) that was compared to warfarin in a population of patients with mechanical valves. The RE-ALIGN trial was ended early after results revealed excess thromboembolic and bleeding events in the dabigatran group. Hence, dabigatran is contraindicated in patients with mechanical heart valves [4]. The remainder DOACs have not been evaluated and, therefore, are not recommended for this indication.

LMWH has a number of advantages over UFH including greater bioavailability, longer duration of effect that allows for once or twice daily dosing and a stronger correlation between dose and anticoagulant response which eliminates the need for laboratory monitoring. However, LMWH’s half-life is prolonged in renal failure since it is primarily excreted by the kidney [5]. In addition, uremia increases the risk of bleeding. Most trials involving LMWH excluded patients with a creatinine clearance (CrCl) of less than or equal to 30 mL/min. There has been a meta-analysis evaluating the bleeding risk in patients with renal insufficiency on LMWH. The patients with renal disease on enoxaparin had increased anti-factor Xa activity than those without renal disease. Data for newer LMWH agents including dalteparin and tinzaparin was insufficient to derive recommendations in renal disease [6]. They may be promising in the future, however, as they do not seem to bioaccumulate in patients with renal insufficiency.

LMWH has been studied in comparison to UFH when administered for the prevention of deep vein thrombosis (DVT) or pulmonary embolism (PE) in patients with ESRD. Results demonstrated no difference in efficacy or significant bleeding between the two agents [7]. This indicates that LMWH may be safe in ESRD patients at low doses. LMWH for treatment of a DVT or PE in ESRD has not been studied extensively. Anti-factor Xa levels do not need to be monitored if LMWH is prescribed for thromboprophylaxis. With therapeutic dosing, biological monitoring of anti-factor Xa activity seems to be necessary. Mechanical heart valve anticoagulation would require therapeutic dosing and, therefore, anti-factor Xa activity monitoring if LMWH is used. Although anti-factor Xa activity is the gold standard marker for plasma concentration monitoring of LMWH, it is not well correlated with the incidence of hemorrhage. Unfortunately, there are no guidelines for anti-factor Xa monitoring with LMWH [3].

UFH can be used in renal failure since it lacks substantial renal metabolism. Nonetheless, it is difficult to use long-term in an outpatient setting due to its narrow therapeutic window of adequate anticoagulation. Maintaining activated partial thromboplastin time (aPTT) in a therapeutic range is challenging with aPTT being outside the therapeutic range 60–70% of measurements despite close monitoring and proper dose titration [8]. There are studies involving pregnant women with mechanical heart valves who developed more thromboembolic phenomenon when anticoagulated with UFH alone in comparison to warfarin throughout their pregnancy [9, 10]. Long-term UFH also leads to adverse effects on bone mineral density and patients should receive calcium supplementation to reduce the development of osteoporosis. Calcium supplementation also exacerbates calciphylaxis and should not be started in those with the condition.

**Fig. 1:**
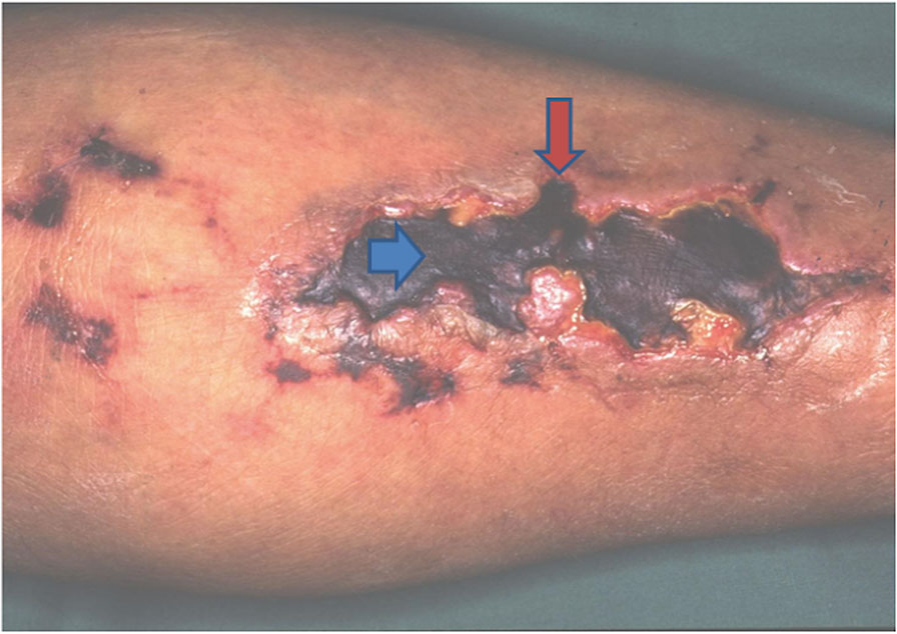
This demonstrates a typical skin lesion secondary to calciphylaxis. Note the necrosis of the ulceration and the development of eschar. The red arrow points to the border of the ulceration. The blue arrow demonstrates the eschar. Printed under the permission of Dermnetnz.org

**Fig. 2:**
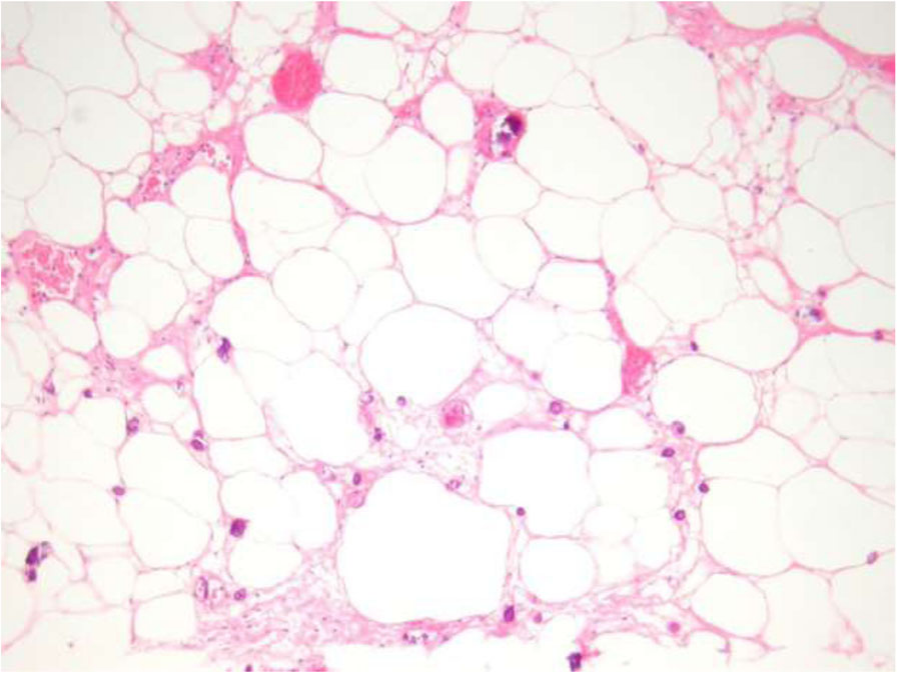
A hematoxylin and eosin stain of a calciphylaxis ulceration revealing calcification of the small and medium-sized vessels (blue arrow). There is evidence of perivascular and interstitial calcifications (green arrow). Hemorrhagic lipid necrosis of the adipocytes may also be seen as presented in this image (red arrow). Printed under the permission of Dermnetnz.org

Calciphylaxis is a rare and life-threatening disorder consistent of skin ischemia and necrosis. It mainly occurs in ESRD patients and the prognosis is very poor. It is characterized by skin lesions that begin as painful nodules and progress to necrotic ulcers that often become superinfected (Fig. 1). They tend to develop in areas with the most adipose tissue as was seen in our patient who developed lesions in his thigh. The diagnosis is confirmed by biopsy that reveals arteriolar occlusion and calcification with no vasculitic changes (Fig. 2). The optimal management is not known but involves wound care, treating hyperphosphatemia and secondary hyperparathyroidism and administering sodium thiosulfate. Sodium thiosulfate has been shown to resolve or improve the calciphylactic lesions when studied retrospectively [11]. Most importantly, medications that contribute to calciphylaxis should be discontinued if possible. These medications include vitamin D, calcium supplementation, warfarin, glucocorticoids and iron.

Despite the multiple risk factors associated with the development of calciphylaxis, the pathogenesis is poorly understood. Hayashi et al. [12] revealed a 10.1-fold increased risk of calciphylaxis with warfarin use. Other studies demonstrate a correlation between warfarin use and the development of vascular calcifications. Warfarin produces an imbalance between pro and anti-calcification factors. It is thought to inhibit a matrix G1A protein (MGP) that prevents tissue mineralization. This vitamin K dependent protein consists of 84 amino acids and is activated by the carboxylation of glutamine residues. Extensive vascular calcification was demonstrated when the protein was inhibited in rat models [13].

Theoretically, vitamin K supplementation should suppress calcification. The two subtypes of vitamin K are vitamin K1 (phylloquinone) and K2 (menaquinone). Vitamin K1 is found in leafy, green vegetables and plant oils while vitamin K2 sources are egg yolks, lard, butter and animal-based foods. Vitamin K2 consists of nine short and long-chain menaquinones with MK-4 being the most important one. K vitamins serve as cofactors for the enzyme gamma-glutamyl carboxylase. This enzyme is involved in the vitamin-K dependent carboxylation of G1A domain of G1A proteins. G1A proteins are found in factors II, VII, IX and X and vitamin K1 aids in activating these factors with carboxylation. Vitamin K2 activates MGP through the same mechanism and this prevents calcium deposition in the lining of blood vessel walls [13].

Since both subtypes play a role as cofactors for gamma-glutamyl carboxylase, they may both be involved in the reduction of vascular calcification. Observational studies based on dietary intake suggest K2 may be more likely to protect against vascular calcification than K1. However, one intervention study where subjects were given K1 supplementation and vascular calcification was measured demonstrated association between the supplementation and reduced coronary artery calcification (CAC) progression in 388 older men and women free of cardiovascular disease [14].

The risk and benefits of the continuation of warfarin should be weighed against the risk of ulcerations and necrosis. The benefits of continuing warfarin in our patient exceeded the risk of discontinuation in the setting of two mechanical heart valves, chronic AF and no other feasible option for anticoagulation. The patient did expire and the question of whether exchanging warfarin for UFH or LMWH would have been better options still arises. This remains a dilemma without a straightforward answer.

## Conclusion

Antithrombotic therapy in patients with mechanical heart valves continues to be a serious challenge. The choices are limited and problems may appear when patients have contraindications to the currently approved agents. In ESRD patients suffering from calciphylaxis, warfarin and enoxaparin cannot be used as warfarin intensifies the disease burden and enoxaparin at therapeutic dosing was not studied in patients with low CrCl. Newer LMWH agents seem promising since they do not bioaccumulate in renal patients. Further studies involving new LMWH agents and patients with CrCl < 30 mL/min are needed to determine the utility of LMWH in ESRD. Research exploring the effect of other DOACs on the anticoagulation of mechanical heart valves are imperative for determining the optimal antithrombotic regimen in the future.

## Abbreviations

AF: Atrial fibrillation
aPTT: Activated partial thromboplastin time
CAC: Coronary artery calcium
CrCl: Creatinine clearance
DOAC: Direct oral anticoagulant
DVT: Deep vein thrombosis
ESRD: End-stage renal disease
FDA: Food and drug administration
LMWH: Low molecular weight heparin
MGP: Matrix G1A protein
PE: Pulmonary embolism
RHD: Rheumatic heart disease
UFH: Unfractionated heparin
VKA: Vitamin K antagonist
